# Osthole suppresses prostate cancer progression by modulating PRLR and the JAK2/STAT3 signaling axis

**DOI:** 10.3389/fonc.2025.1630181

**Published:** 2025-09-05

**Authors:** Linjun Yan, Jiaqi Mei, Yuanqiao He, Qi Cui, Xiaohong Wang, Feng Lv, Keyue Cao, Yuanjian Shao

**Affiliations:** ^1^ School of Environmental and Biological Engineering, Nantong College of Science and Technology, Nantong, Jiangsu, China; ^2^ Department of Hematology, The Second Affiliated Hospital, Jiangxi Medical College, Nanchang University, Nanchang, Jiangxi, China; ^3^ Center of Laboratory Animal Science, Nanchang University, Nanchang, Jiangxi, China; ^4^ Key Laboratory of New Drug Evaluation and Transformation of Jiangxi Province, Nanchang, Jiangxi, China; ^5^ Nanchang Royo Biotech Co., Ltd., Nanchang, Jiangxi, China

**Keywords:** osthole, prostate cancer, JAK2/STAT3 pathway, network pharmacology, PRLR

## Abstract

**Background:**

Prostate cancer is a common malignancy in men with limited effective treatment options, highlighting an urgent need for novel therapeutic approaches. Osthole, a natural coumarin compound with antitumor properties, has shown potential in targeting various cancers.

**Methods:**

We conducted the study using a combination of network pharmacology, *in vitro* assays, and *in vivo* experiments. First, network pharmacology was used to predict the potential targets of Osthole, identifying 68 targets shared with prostate cancer, including AKT1, TNF, IL6, STAT3, and CTNNB1. Subsequently, we confirmed these targets and assessed the effects of Osthole on cell proliferation, migration, and apoptosis using the Cell Counting Kit-8 (CCK-8) and transwell invasion assays. Meanwhile, molecular docking and western blot analysis were employed to analyze molecular interactions and protein expression levels.

**Results:**

Our findings revealed that Osthole significantly inhibited prostate cancer cell proliferation and migration in a dose-dependent manner and reduced tumor volume in *in vivo* assays. Western blot analysis indicated that Osthole downregulated PRLR expression and decreased the phosphorylation of JAK2 and STAT3, suggesting the inhibition of the JAK2/STAT3 signaling pathway.

**Conclusion:**

These results collectively highlight the therapeutic potential of Osthole in targeting prostate cancer cells through PRLR and modulating the JAK2/STAT3 signaling pathway, warranting further clinical exploration.

## 1 Introduction

Prostate cancer, which originates in the prostate gland in males, is recognized as one of the most prevalent malignancies within the male urogenital system (1). Globally, prostate cancer ranks third in occurrence and sixth in mortality among men (2). Prostate cancer is predominantly treated with surgical resection, castration, and radiochemotherapy (3). However, owing to the limited effectiveness of conventional therapeutic approaches in improving the survival of patients with poor prognoses, there is an urgent need to develop novel treatment modalities.

Cnidium monnieri extract is a herbal extract derived from medicinal plants such as Cnidium monnieri and Angelica sinensis, containing a variety of bioactive components. Osthole, on the other hand, is a specific naturally occurring compound that is one of the key active ingredients in Cnidium monnieri extract. This compound, classified within the coumarin class of chemical compounds, exhibits a diverse array of biological activities, including antitumor, anti-inflammatory, neuroprotective, osteogenic, cardiovascular protective, antimicrobial, and antiparasitic effects (4). Notably, osthole has been characterized to possess favorable pharmacological properties: it shows relatively low toxicity with a good safety profile within therapeutic doses, as supported by preclinical studies; its lipophilic nature leads to limited water solubility, though this can be improved through formulation strategies like nanocarrier-based delivery systems; and its metabolism primarily occurs in the liver via cytochrome P450 enzymes, with well-defined metabolic pathways and elimination routes (5). There is documented evidence in the literature that Osthole has been utilized for the treatment of various cancers, including gastric, bladder, and breast cancers (6–8). Additionally, only a limited number of studies have documented the inhibitory effects of Osthole on prostate cancer *in vitro* (9). However, research on its *in vivo* effects remains limited. These findings collectively highlight Osthole’s potential as an anticancer drug candidate, particularly due to its multi-targeted actions and favorable safety profile, which distinguish it from many conventional chemotherapeutics. However, further *in vivo* studies and clinical trials are warranted to fully validate its therapeutic efficacy and establish optimal administration protocols.

The prolactin receptor (PRLR), a key member of the growth hormone receptor family, has been closely associated with hormone-dependent cancers such as breast and ovarian cancers (10). However, its specific role and underlying mechanisms in prostate cancer remain poorly understood. PRLR is known to activate multiple downstream signaling pathways, among which the JAK2/STAT3 pathway plays a crucial role in tumor cell proliferation, immune evasion, and therapeutic resistance (11). Investigating the regulatory mechanisms of PRLR and its downstream JAK2/STAT3 signaling axis in prostate cancer may provide a scientific foundation for the development of novel targeted therapies. Previous studies have demonstrated that Osthole can regulate pathways such as PI3K/Akt and MAPK, thereby inhibiting tumor growth in gastric and bladder cancers (12). However, whether Osthole exerts its anticancer effects in prostate cancer by modulating PRLR and the JAK2/STAT3 signaling axis has not been thoroughly investigated. The JAK2/STAT3 signaling pathway plays a crucial role in various cancers, including prostate cancer. Its abnormal activation is closely associated with tumor cell proliferation, survival, immune evasion, and therapeutic resistance. In prostate cancer, sustained activation of the JAK2/STAT3 pathway is linked to tumor progression and poor patient prognosis (13). Research indicates that inhibiting this pathway can reduce tumor cell proliferation and invasion and enhance chemotherapeutic drug sensitivity (14). Thus, targeting the JAK2/STAT3 pathway offers a promising strategy for prostate cancer therapy.

Given the pivotal role of PRLR and JAK2/STAT3 signaling in hormone - dependent cancers, exploring their regulatory mechanisms in prostate cancer is vital for developing new treatments. Osthole, a natural compound, has demonstrated anticancer activity by modulating multiple pathways, including PI3K/Akt and MAPK (15). However, whether Osthole exerts its effects via PRLR and JAK2/STAT3 signaling in prostate cancer remains unclear. Therefore, in this study, we utilized network pharmacology to predict the common targets of Osthole and prostate cancer, which were subsequently validated through a series of *in vivo* and *in vitro* experiments. This study could potentially contribute to the development of novel therapeutic agents for the treatment of prostate cancer.

## 2 Materials and methods

### 2.1 Experimental materials

Prostate cancer cell lines RM1, 22RV1, PC-3, and DU145, along with RPMI 1640 culture medium and other reagents, were procured from Fuheng Biological Technology Co., Ltd. RPMI 1640 culture medium was acquired from Tianjin Haoyang Biological Products Technology Co., Ltd and Osthole (molecular weight: 244.29; purity: 99%) was obtained from Shanghai Yien Chemical Technology Co., Ltd., For the solvents used to dissolve osthole: Dimethyl Sulfoxide (DMSO) was used for *in vitro* studies, while corn oil was used for *in vivo* studies (referenced to MCE official website【https://www.medchemexpress.cn/Osthole.html】. Details regarding these solvents and whether the same solvent was used as vehicle control in *in vitro* and *in vivo* studies are provided in the reference (16). For *in vitro* studies, Osthole was dissolved in DMSO (final concentration ≤0.1%, matched in controls). For *in vivo* administration, it was diluted in pure corn oil (vehicle control: corn oil alone).

### 2.2 Network pharmacological analysis

#### 2.2.1 Selection of osthole targets

Three-dimensional structural information of the compound was retrieved from the PubChem database (https://pubchem.ncbi.nlm.nih.gov/). Target proteins of the compound were identified using the Swiss Target Prediction (STP) database (http://www.swisstargetprediction.ch/) with a probability threshold greater than zero. Additionally, the BATMAN-TCM2.0 database (http://bionet.ncpsb.org/batman-tcm/) was used to filter target proteins with a cutoff value exceeding 0.85. The resulting target proteins were consolidated by eliminating duplicates to yield a list of potential target proteins.

#### 2.2.2 Collection of prostate cancer targets

Genes linked to prostatic cancer were identified by searching the term ‘prostatic cancer’ in DisGeNET(https://www.disgenet.org/), Genecards(https://www.genecards.org/), and OMIM databases(https://omim.org/) with specific filters: a GDA score >0.1 DisGeNET, a relevance score >10 in Genecards, and relevant gene entries in OMIM. After removing duplicates, the compiled gene list was intersected with the target proteins of the compound to identify the shared targets.

#### 2.2.3 Construction of protein-protein interaction

The intersecting target dataset was imported into the STRING database (https://string-db.org/) with *Homo sapiens* selected as the *species*, and the minimum required interaction score was set to 0.4. The ‘string_interactions_short.tsv’ file was then downloaded and imported into Cytoscape 3.7.2 for network visualization. Network topology analysis was performed using the CytoNCA plugin.

#### 2.2.4 Construction of drug-compound-target network diagram

The intersection of disease and compound targets identified a set of genes potentially responsible for therapeutic effects. Data corresponding to these genes were extracted from the drug database and compiled into a network, which was then saved as ‘network.xlsx’. Subsequently, this network data file was imported into the Cytoscape 3.7.2 to visualize the network.

#### 2.2.5 Pathway analysis of targets

Enrichment analysis for the intersecting targets was performed using the R packages ‘ClusterProfiler’, ‘org.Hs.eg.Db’, and ‘ggplot2’. Gene annotation was sourced from the ‘ package. The analysis utilized an adjusted p-value threshold of 0.05 and a q-value threshold of 0.05 to determine statistical significance.

#### 2.2.6 Molecular docking

Top-ranked macromolecules were docked with small molecules from the PubChem database using protein structures from the AlphaFold Database. Protein structures were prepared using AutoDockTools 5.6, and small molecules were processed using Open Babel and AutoDock. Docking was performed using AutoDock, and the results were visualized using PyMOL.

### 2.3 Cell functional assays

#### 2.3.1 Cell proliferation assay

22RV1, PC-3, DU145, and RM1 cell lines were cultured in RPMI-1640 medium supplemented with 10% FBS. The cells were then incubated in a humidified incubator at 37 °C with 5% CO_2_. Cells in the logarithmic growth phase at approximately 90% confluence were treated with trypsin, seeded at 10,000 cells per well in a 96-well plate, and cultured for 24 hours. Subsequently, the cells were treated with Osthole or DMSO and incubated for 48 hours. After treatment, CCK-8 was added, and the cells were incubated for 1–4 hours. Absorbance was measured at 450 nm using an enzyme-linked microplate reader. Each experiment was performed in triplicate, and the experiment was repeated at least twice. Data are presented as mean ± standard deviation.

#### 2.3.2 Transwell invasion assay

Matrigel (BD Biosciences, San Jose, CA, USA) was diluted at a 1:3 ratio in serum-free RPMI-1640, and 50 μL of the diluted Matrigel was used to pre-coat the Transwell inserts. RM1 cells were treated with either 0 μM (control group) or 100 μM Osthole for 48 hours. After treatment, the cells were collected and resuspended in serum-free DMEM at a density of 1×10^5^ cells/mL, and 500 μL of this cell suspension was added to the upper chamber. Subsequently, 600 μL of RPMI-1640 containing 10% FBS was added to the lower chamber. The RM1 cells were incubated at 37 °C with 5% CO_2_ for 24 hours to allow invasion. Invasive cells were fixed with 4% paraformaldehyde for 15 minutes at room temperature and then stained with 0.1% crystal violet for 10 minutes at room temperature. Finally, the invading cells were photographed and counted under an inverted microscope, with five random non-overlapping fields selected for cell counting.

#### 2.3.3 Wound healing assay

The wound healing assay evaluated migration of RM1 cells. Log-phase cells were trypsinized (0.25%), resuspended in 10% FBS medium, counted via hemocytometer, and seeded at 50–100×10^4^ cells/well in 6-well plates. Cultured at 37 °C in 5% CO_2_ to 95%–100% confluence, scratches were made with a 200-μl tip (ruler-guided) to create 2–3 vertical/horizontal wounds per well, followed by 2–3 PBS washes to remove detached cells.

Cells were treated with 0, 100, 200 μM Osthole (2 ml/well). Initial wound images at 0 h were captured at 20× objective for 5 fixed fields/well, with subsequent imaging at 0, 24 h (or when control wound closure >50%) to monitor migration.

### 2.4 Tumor inoculation and monitoring

RM1 and 22RV1 cell suspensions were prepared such that each mouse received an injection of 3×10^6^ cells (in 0.1 mL PBS); these suspensions were separately injected subcutaneously into the right scapular region of mice to induce tumor growth. Specifically, RM1 cells were injected subcutaneously into C57 mice, while 22RV1 cells were injected subcutaneously into BALB/c nude mice. Prior to inoculation, the skin at the injection site was prepared using a hair shaver (for haired mice) and disinfected with iodophor to reduce the risk of infection. No anesthesia was administered during inoculation due to the minimally invasive nature of subcutaneous injection; however, procedures were performed rapidly to minimize animal stress and discomfort. Tumor volume was monitored and measured biweekly with calipers, allowing the tumors to reach an approximate size of 100 mm^3^ before proceeding with experimental manipulation. Once the tumors reached 100 mm^3^, the mice were randomly assigned to two groups, each consisting of five animals: a treatment group and a control group. For the RM1 model, the control group consisted of 7 mice and the Osthole treatment group consisted of 6 mice. For the 22RV1 model, both the control and treatment groups consisted of 5 mice each. Osthole group(200 mg/kg, IP, once a week for three weeks). The control group was injected with an equal volume of Corn oil. The drug concentration refers to reference (17).

Tumor volume and weight were measured twice weekly, with observations of tumor morphology and ulceration status before and after each treatment. Tumor volume was expressed in cubic millimeters (mm^3^), calculated using the formula: (V = 0.5×a×b^2^), where a and b represent the longest and shortest diameters of the tumor, respectively. Tumor weight was assessed at the experimental endpoint (after treatment completion) by excising and weighing tumors following euthanasia. Before and after each treatment, detailed observations of tumor morphology and ulceration status were conducted to identify signs of distress. Prophylactic antibiotic treatment (systemic antibiotics for 3 days or antibiotic jelly for 5 days) was administered post-inoculation to prevent infection at the injection site and alleviate potential discomfort.

### 2.5 Animal ethics and experimental methods

In the *in vivo* studies using RM1 and 22RV1 mouse models, strict humane euthanasia endpoints were defined: tumor volume exceeding 1500 mm³; the control group’s average tumor volume reaching 500 mm³ at 21 days post-drug intervention; tumor ulceration/necrosis; or body weight loss exceeding 20% of initial weight (accounting for tumor burden). Experiments utilized two mouse strains—C57 mice (6–8 weeks old, ~20 g) for the RM1 model and BALB/c nude mice (5 weeks old, ~20 g) for the 22RV1 model—with study timelines of August 4, 2022–December 20, 2022 (RM1) and November 24, 2023–March 20, 2024 (22RV1), involving 23 mice total. At study completion, mice were euthanized via CO_2_ inhalation following IACUC-approved protocols (Approval Nos.: RYE2022080301, RYE2023112501). Throughout the study, twice-weekly monitoring included tumor volumes, body weights, and health parameters (behavior, coat condition, gait) to detect distress; tissue processing excluded necrotic/non-tumor regions using sterile techniques, and prophylactic antibiotics (systemic: 3 days; topical jelly: 5 days post-inoculation) minimized infection and procedural suffering. Protocols, reviewed and approved by the IACUC, prioritized animal welfare via sterile/rapid procedures, strict euthanasia endpoints to avoid prolonged distress, and regular health assessments to maintain well-being.

### 2.6 Bioinformatics analysis of PRLR, JAK2, and STAT3 in prostate cancer using TCGA data

RNA-seq data and clinical information for prostate cancer patients were obtained from the TCGA-PRAD dataset, including 496 tumor samples and 52 normal samples. Differential gene expression analysis was performed using the “limma” R package, with |log2FC| ≥ 1 and adjusted p-value < 0.01 as significance thresholds. Kaplan-Meier survival analysis was conducted for PRLR, JAK2, and STAT3 using the “survival” and “survminer” R packages, categorizing patients into high- and low-expression groups based on the median expression value, and survival differences were assessed via log-rank tests. Gene Ontology (GO) and KEGG pathway enrichment analyses of differentially expressed genes were performed using “clusterProfiler”, with adjusted p-value < 0.05 as the cutoff. Gene Set Enrichment Analysis (GSEA) for PRLR, JAK2, and STAT3 was conducted with the “fgsea” and “enrichplot” R packages to explore associated pathways. All analyses and visualizations were performed in R (version 4.2.0) with additional packages including “ggplot2”, “pheatmap”, and “AnnotationDbi”.

### 2.7 Western blot analysis of RM1 cells

RM1 cells were lysed for protein extraction, and equal amounts of protein were separated by SDS-PAGE and transferred to PVDF membranes. After blocking, the membranes were incubated overnight at 4 °C with primary antibodies against JAK2 (Immunoway, China; Cat# ^[YM8330]^; Rabbit mAb; 1:5000), p-JAK2 (Immunoway, China; Cat# ^[YP0155]^; Rabbit mAb; 1:1000), STAT3 (Immunoway, China; Cat# ^[YM8325]^; Rabbit mAb; 1:2000), p-STAT3 (Immunoway, China; Cat# ^[YM8680]^; Rabbit mAb; 1:5000), and GAPDH (Immunoway, China; Cat# ^[YM8394]^; Rabbit mAb; 1:30000). The membranes were then incubated with HRP-conjugated secondary antibodies (Immunoway, China; Cat# ^[RS0002]^; 1:10000) at room temperature for 1 h. Protein bands were visualized using Enhanced Chemiluminescence, quantified by densitometry with ImageJ software, and normalized to GAPDH.

### 2.8 Molecular dynamics simulation

Molecular dynamics (MD) simulations were performed using Gromacs 2022.3 software to investigate the interaction and stability of the PRLR-Osthol complex. The initial structure of the complex was prepared from the molecular docking results. The protein was parameterized using the Amber99sb-ildn force field, and the small molecule Osthole was preprocessed with AmberTools22 to assign the GAFF force field. Gaussian 16W was employed for hydrogenation of the small molecule and calculation of RESP charges, which were incorporated into the system topology file.

The simulation system was solvated in a cubic periodic boundary box with a 1.2 nm distance from the protein surface using the TIP3P water model. Na^+^ ions were added to neutralize the total charge of the system. Prior to production simulation, energy minimization was performed using the steepest descent method to eliminate steric clashes. Subsequently, the system was equilibrated under the isothermal-isochoric (NVT) ensemble for 100 ps (100,000 steps) to stabilize the temperature at 300 K, followed by isothermal-isobaric (NPT) ensemble equilibration for another 100 ps (100,000 steps) to maintain a constant pressure of 1 Bar, with a coupling constant of 0.1 ps for both equilibration steps.

The production MD simulation was conducted for 100 ns with a time step of 2 fs, resulting in 500,0000 integration steps. Temperature was controlled using the velocity rescaling thermostat, and pressure was maintained with the Parrinello-Rahman barostat. All bonds involving hydrogen atoms were constrained using the LINCS algorithm.

Trajectory analysis was performed using Gromacs built-in tools. Key parameters including root-mean-square deviation (RMSD) of the protein backbone and ligand, root-mean-square fluctuation (RMSF) of residues, radius of gyration (Rg), and solvent-accessible surface area (SASA) were calculated to evaluate complex stability and conformational changes. Hydrogen bond occupancy was analyzed to assess intermolecular interactions. Binding free energy was computed using the molecular mechanics/generalized Born surface area (MM/GBSA) method, with contributions from van der Waals interactions, electrostatic interactions, and solvation energies decomposed to understand the binding mechanism.

All figures were generated using GraphPad Prism, and molecular visualizations were performed using PyMOL software.

### 2.9 Statistical analysis

All calculations were performed using Prism 6, and the data are presented as mean ± standard deviation (SD). Multiple group comparisons were made using two-way ANOVA, with a p-value of less than 0.05 considered statistically significant.

## 3 Results

### 3.1 Network pharmacology analysis of osthole targets and pathways in prostate cancer

**Figure 1:**
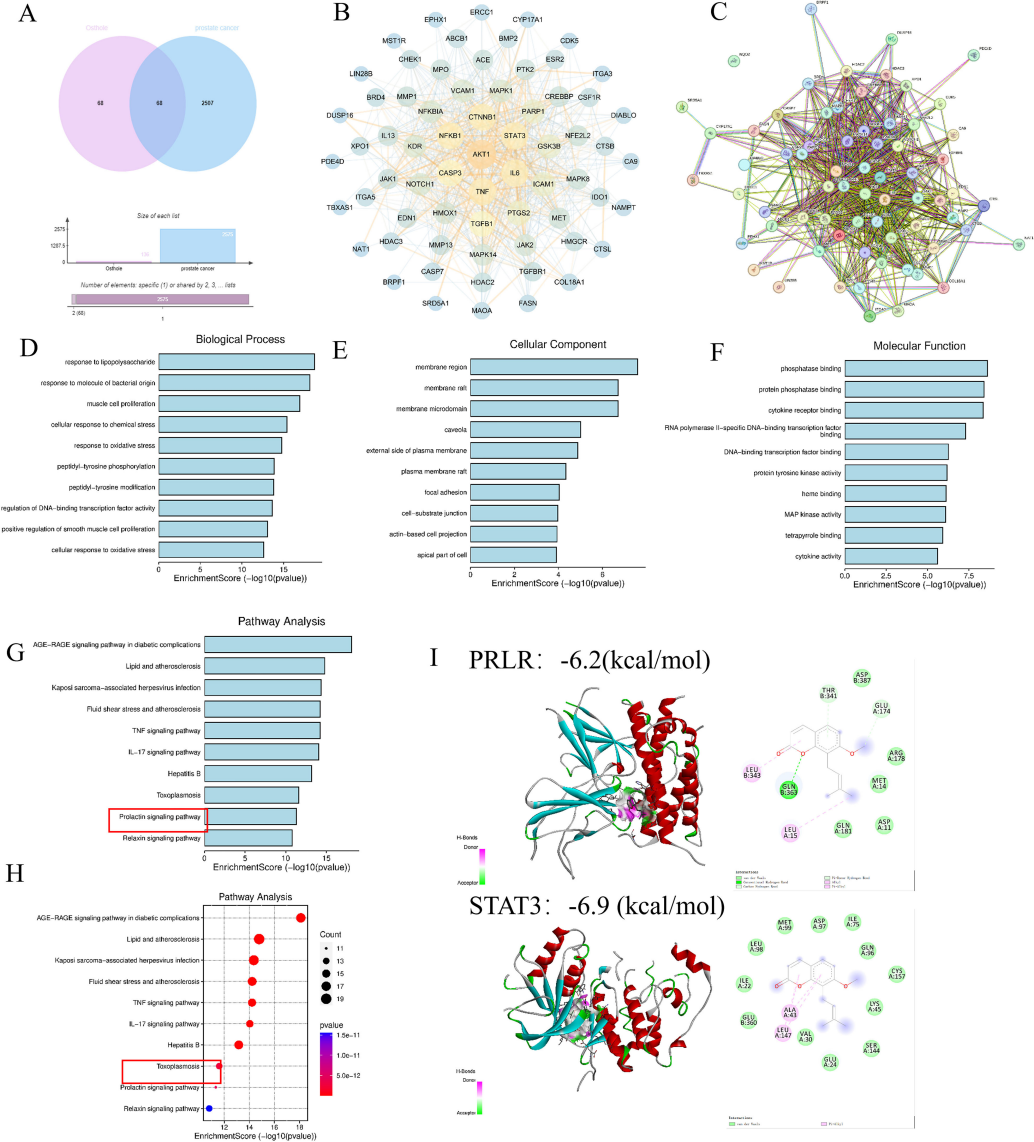
Network pharmacology analysis. **(A, B)** Analysis of databases revealed 68 shared targets for Osthole in prostate cancer. **(C)** PPI network analysis using STRING database identified AKT1, TNF, IL6, STAT3, and CTNNB1 as possible therapeutic targets of Osthole in prostate cancer. **(D-F)** Intersection and GO analysis revealed Osthole’s association with critical prostate cancer-related pathways and functions. **(G, H)** Kyoto Encyclopedia of Genes and Genomes (KEGG) analysis revealed the involvement of Osthole in the PRL pathway and other significant cancer-related pathways. **(I)** Molecular docking results of PRLR and STAT3 with Osthole. The panel displays two target proteins (PRLR, top; STAT3, bottom) docked with the small molecule Osthole. For each protein:3D structure: Shows the protein’s secondary structure (e.g., α-helices in red/cyan) with Osthole (colored spheres/sticks) bound in its active pocket. 2D interaction diagram: Adjacent to each 3D structure, illustrates Osthole’s direct interactions with key amino acid residues of the protein. Binding energy: −6.2 kcal/mol (PRLR) and −6.9 kcal/mol (STAT3). More negative values indicate stronger binding affinity.

Potential targets of Osthole were predicted using the Swiss Target Prediction and BATMAN-TCM2.0 databases, and 68 valid targets were identified after excluding the ineffective components. Concurrently, 2507 prostate cancer-related targets were filtered from the DisGeNET, Genecards, and OMIM databases. Venn diagram analysis revealed 68 common targets between Osthole and prostate cancer (**Figure 1A**). Subsequently, PPI network analysis was conducted on these targets using the STRING database, which suggested that genes such as AKT1, TNF, IL6, STAT3, and CTNNB1 might be key targets for Osthole in the treatment of prostate cancer (**Figures 1B, C**).

Furthermore, an intersection approach was employed to identify disease-related targets from the drug data, which were then analyzed using Cytoscape 3.7.2. Gene Ontology (GO) enrichment analysis yielded a total of 3993 results, including 3469 biological process (BP) terms primarily associated with lipopolysaccharide, response to molecules of bacterial origin, and muscle cell proliferation; 191 cellular component (CC) terms related to membrane regions, membrane rafts, and membrane microdomains; and 371 molecular function (MF) terms involving phosphatase binding, protein phosphatase binding, and cytokine receptor binding (**Figures 1D–F**). The Kyoto Encyclopedia of Genes and Genomes (KEGG) enrichment analysis identified 226 pathways, predominantly involving the advanced glycation end-product receptor for advanced glycation end-product signaling pathway in diabetic complications and lipid and atherosclerosis, suggesting that Osthole may intervene in prostate cancer through these pathways (*P* < 0.05) (**Figures 1G, H**).

Additionally, molecular docking with a binding energy of less than -1 kcal/mol indicates active binding, while a value less than -5 kcal/mol suggests a stronger binding affinity. The molecular docking results demonstrated that STAT3 and PRLR exhibit favorable binding activities with Osthole, with binding energies of -6.9 and -6.2 kcal/mol, respectively, indicating a strong affinity of these proteins for Osthole (**Figure 1I**).

### 3.2 Structural stability and binding affinity characteristics of the PRLR-osthole complex revealed

To investigate the interaction and complex stability between the small molecule Osthole and the PRLR protein, a 100 ns molecular dynamics (MD) simulation was performed. Comprehensive evaluations of the complex stability and binding characteristics were conducted by integrating the results of root-mean-square deviation (RMSD), radius of gyration (Rg), solvent-accessible surface area (SASA), number of hydrogen bonds, root-mean-square fluctuation (RMSF), and molecular mechanics/generalized Born surface area (MM/GBSA) binding free energy calculations.

**Figure 2:**
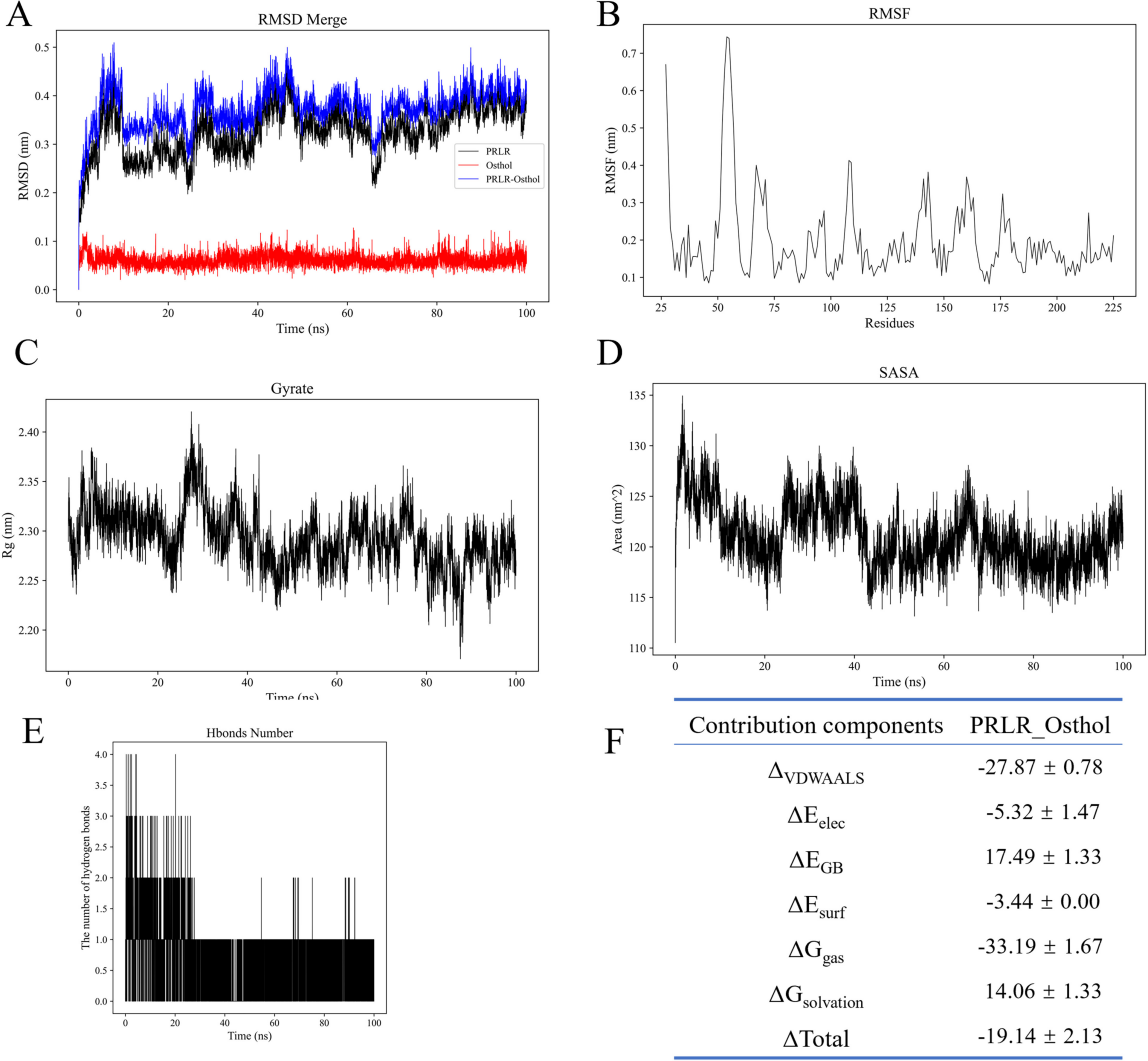
Molecular dynamics simulation analyses of PRLR-Osthole complex stability and binding properties. **(A)** Root-mean-square deviation (RMSD) curves for PRLR (black), Osthole (blue), and PRLR-Osthole complex (red) over the 100 ns simulation. The y-axis represents RMSD (nm), and the x-axis represents simulation time (ns). The complex undergoes structural stabilization after ~20 ns, maintaining an average RMSD of 0.37 ± 0.04 nm. **(B)** Root-mean-square fluctuation (RMSF) of PRLR residues. The y-axis denotes RMSF (nm), and the x-axis denotes residue number. Most residues exhibit low flexibility (<0.3 nm), except for local fluctuations in loop regions (e.g., near residue 70), with an overall average RMSF of 0.20 ± 0.11 nm. **(C)** Radius of gyration (Rg) of the PRLR-Osthole complex over time. The y-axis represents Rg (nm), and the x-axis represents simulation time (ns). The Rg fluctuates slightly within the range of 2.20–2.40 nm (average: 2.29 ± 0.03 nm), indicating the compactness of the protein structure. **(D)** Solvent-accessible surface area (SASA) of the PRLR-Osthole complex over time. The y-axis denotes SASA (nm²), and the x-axis denotes simulation time (ns). The complex undergoes SASA contraction within 0–20 ns, stabilizing around 121.12 ± 3.06 nm², suggesting the masking of hydrophobic residues on the protein surface upon Osthole binding. **(E)** Number of hydrogen bonds between PRLR and Osthole over the 100 ns simulation. The y-axis represents the hydrogen bond count, and the x-axis represents simulation time (ns). An average of ~1 hydrogen bond is maintained throughout the simulation, with stabilization after 20 ns, indicating the auxiliary role of hydrogen bonds in maintaining binding stability. **(F)** MM/GBSA binding free energy decomposition for the PRLR-Osthole complex. The table lists the energy components, including ΔVDWAALS (van der Waals interactions), ΔEelec (electrostatic interactions), ΔEGB (polar solvation energy), ΔEsurf (non-polar solvation energy), ΔGgas (gas-phase free energy), ΔGsolvation (solvation free energy), and ΔGtotal (total binding free energy), with their corresponding values (kcal/mol). Favorable van der Waals and electrostatic interactions are the main contributors to binding, while the unfavorable polar solvation energy is offset by other favorable energy terms.

First, as shown in the RMSD curve of the complex (**Figure 2A**), the system underwent certain structural adjustments during the initial phase (approximately 0–15 ns), after which it generally stabilized. The fluctuations diminished after 20 ns, maintaining around 0.37 nm with an average value of 0.37 ± 0.04 nm. This indicates that the conformational state of the system remained relatively stable upon Osthole binding to PRLR, without significant dissociation or large conformational fluctuations.

The compactness of the protein backbone was assessed using the radius of gyration (Rg) (**Figure 2C**). The Rg value fluctuated slightly within the range of 2.20–2.40 nm, with an average of 2.29 ± 0.03 nm, suggesting that the overall protein structure remained compact throughout the simulation, without obvious collapse or excessive expansion.

SASA analysis (**Figure 2D**) revealed that the complex experienced a certain degree of contraction in solvent-accessible surface area within 0–20 ns, after which it stabilized around 121.12 ± 3.06 nm². This trend implies that the binding of the small molecule may lead to the masking of some hydrophobic residues on the protein surface, which contributes to the stability of the overall structure.

Hydrogen bond analysis (**Figure 2E**) demonstrated that an average of approximately 1 hydrogen bond was maintained between the protein and Osthole over the entire simulation period. Although the number of transient hydrogen bonds fluctuated significantly—particularly with marked changes in the first 20 ns—it stabilized thereafter, indicating that hydrogen bonds play an auxiliary role in maintaining binding stability.

RMSF analysis (**Figure 2B**) showed that the overall flexibility of protein residues was low. Except for local fluctuations in individual loop regions (e.g., near residue 70), the fluctuation amplitude of most residues was less than 0.3 nm, with an overall average of 0.20 ± 0.11 nm. This suggests that the protein maintained a well-rigidified structure upon ligand binding.

For the binding free energy, the total free energy of the PRLR-Osthole complex calculated via the MM/GBSA method was ΔG_total = -19.14 ± 2.13 kcal/mol, indicating strong affinity of the complex. Among the energy components, van der Waals interactions (ΔVDWAALS = -27.87 ± 0.78 kcal/mol) and electrostatic interactions (ΔEelec = -5.32 ± 1.47 kcal/mol) were the main favorable contributors to the binding energy. In contrast, the polar solvation energy (ΔEGB = +17.49 ± 1.33 kcal/mol) was an unfavorable term, which was offset by other favorable energy components. Additionally, the gas-phase free energy (ΔGgas = -33.19 ± 1.67 kcal/mol) was much lower than the solvation energy (ΔGsolvation = +14.06 ± 1.33 kcal/mol), further confirming that the complex exhibits extremely strong binding capacity and significant affinity in a solvent-free environment (**Figure 2F**).

### 3.3 Effects of osthole on prostate cancer cell proliferation, migration, and apoptosis

**Figure 3:**
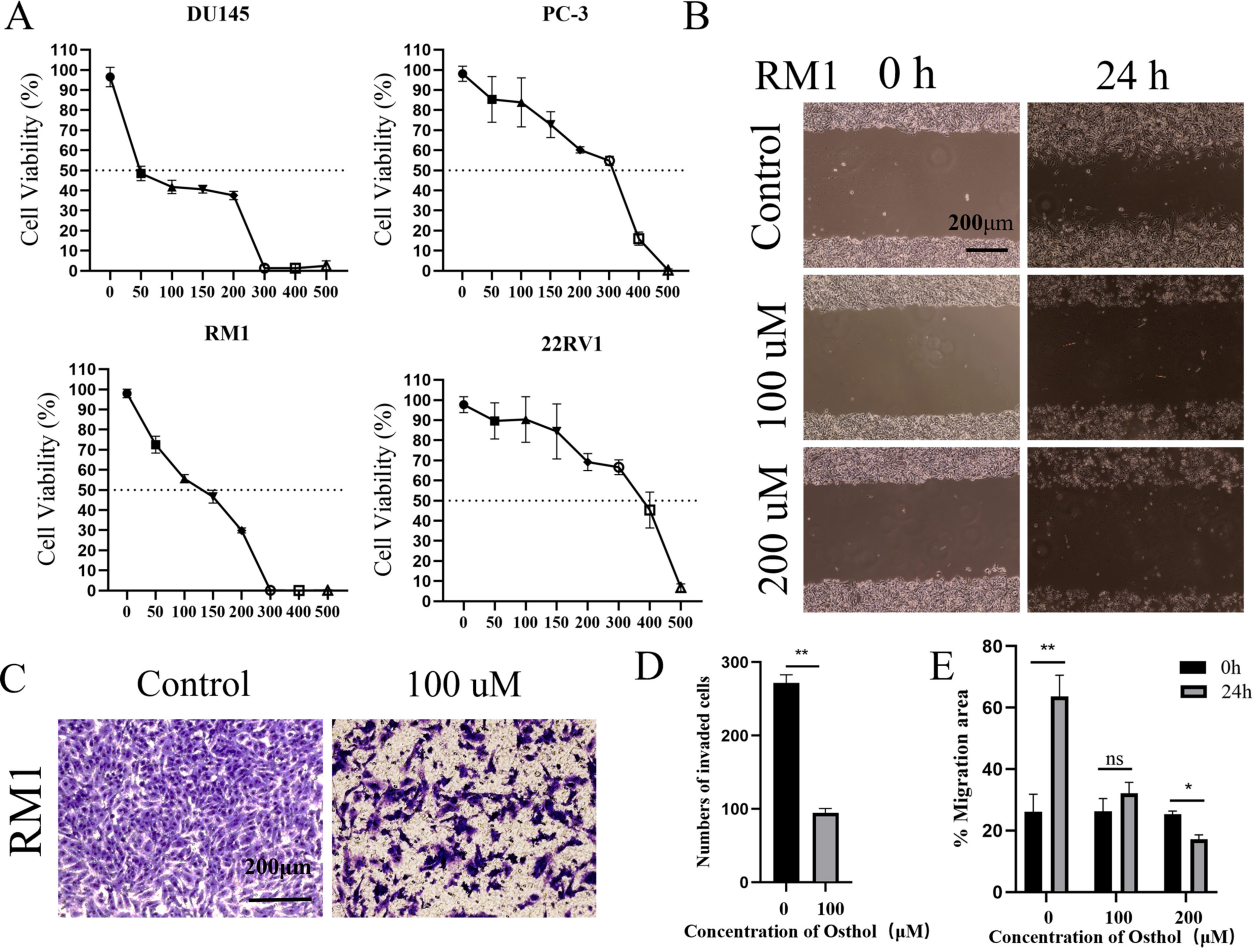
Cell function analysis. **(A)** Osthole induced concentration-dependent cytotoxicity against the prostate cancer cell lines DU145, PC-3, RM1, and 22RV1, yielding half-maximal inhibitory concentration (IC_50_) values of 66.44, 238, 102, and 319.5 µM, respectively. **(B)** Migration of RM1 cells was evaluated via a scratch assay: after culturing cells to confluence, a uniform scratch was created across the monolayer, and cells were treated with varying Osthole concentrations for 24 hours. Cell migration into the scratch area was then monitored. **(C)** Invasiveness of RM1 cells was assessed using a Transwell invasion assay: cells were seeded in Matrigel-coated upper chambers and treated with different Osthole concentrations for 48 hours. Cells that migrated through the Matrigel membrane into the lower chamber were quantified. **(D)** Quantification of the invasive cells from the Transwell invasion assay depicted in **Figure 2C**. The number of cells that migrated through the Matrigel - coated membrane was counted, and the data are presented as the mean ± standard deviation. ** denotes a significant difference (*P* < 0.01) compared to the control group. **(E)** Quantification of cell migration from the scratch assay shown in **Figure 2B**. The percentage of the migration area was calculated, and the data are presented as the mean ± standard deviation. ** indicates a significant difference (*P* < 0.01), * indicates a significant difference *(P <* 0.05*)*, and “ns” (not significant) indicates no significant difference compared to the control group.

We evaluated the effect of Osthole on the proliferation of prostate cancer cell lines using the CCK-8 cell proliferation assay (**Figure 3A**). After treating the cells with 0, 50, 100, 150, 200, 300, 400 and 500 μM of Osthole for 48 hours, we found that it exerted significant cytotoxicity on the 22RV1 and PC-3 cell lines, the half-maximal inhibitory concentration (IC_50_) values for the prostate cancer cell lines DU145, PC-3, RM1, and 22RV1 were determined to be 66.44 μM, 238 μM, 102 μM, and 319.5 μM, respectively. These values indicate that the proliferation of the respective cell lines was inhibited by 50%. Compared with the control group, Osthole-treated cells showed significant suppression of proliferation at all concentrations tested (*P* < 0.05), with cell viability decreasing as Osthole concentration increased, indicating a dose-dependent inhibitory effect on prostate cancer cell proliferation.

Additionally, the effect of Osthole on the migratory ability of prostate cancer cells was assessed using the scratch assay (**Figures 3B, E**). Cells were treated with Osthole at 100 μM and 200 μM, and 24 h post-scratch, the Osthole-treated groups showed significantly reduced cell migration distance compared to the control (*P* < 0.05). These results suggest that Osthole effectively inhibits prostate cancer cell migration in a dose-dependent manner.

The Control group showed a high number of cells that invaded the Matrigel-coated membrane, indicating a strong invasive capability. The cells were densely packed and uniformly distributed, suggesting their active migration and invasion. The Osthole group showed a marked decrease in the number of invading cells. The cells appeared less dense and more scattered (**Figures 3C, D**), indicating that Osthole significantly impaired their invasive ability. This reduction in cell invasion suggests that Osthole effectively inhibits the cellular mechanisms involved in invasion and metastasis.

### 3.4 *In vivo* tumor growth inhibition by osthole in prostate cancer

**Figure 4:**
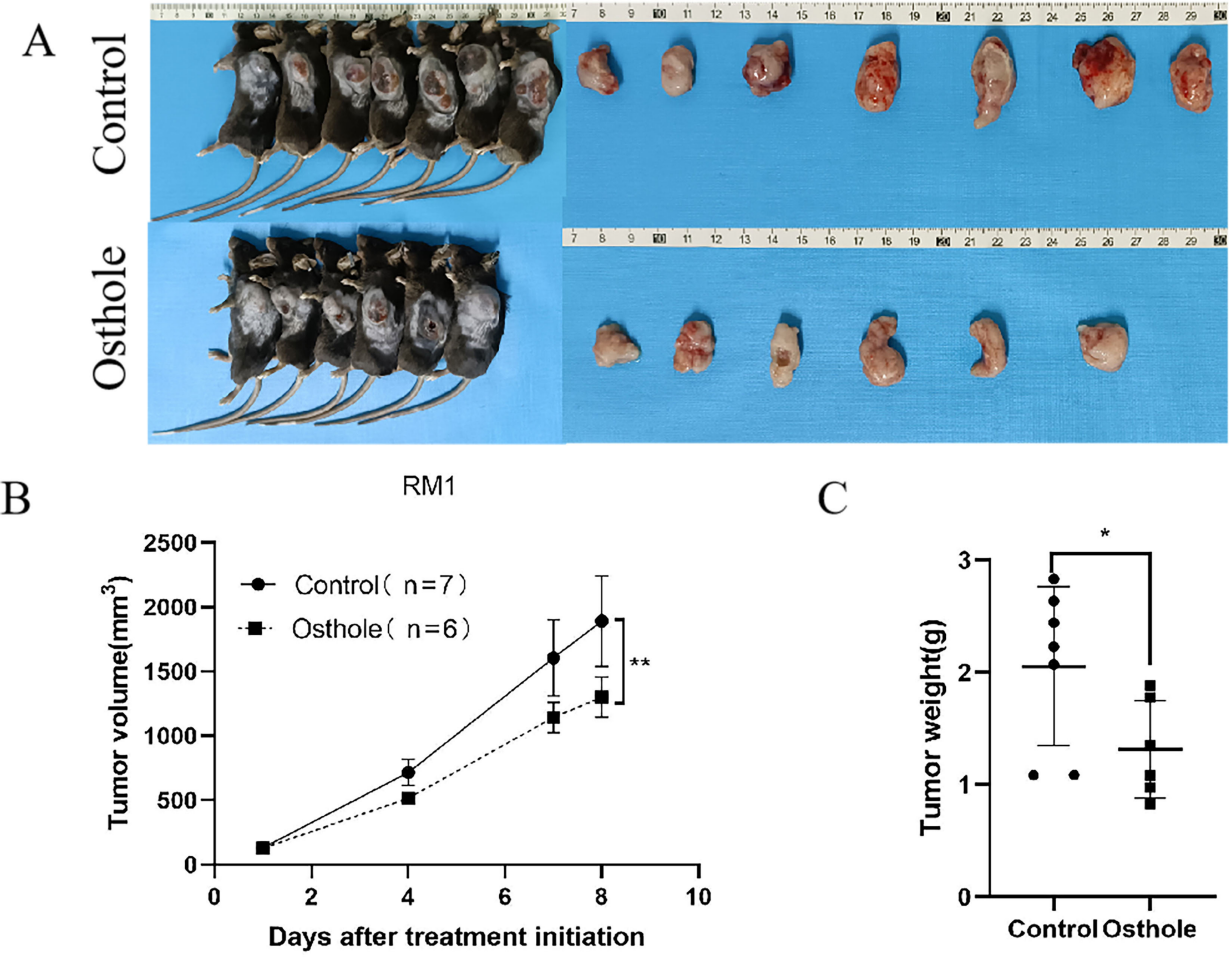
Tumor growth and weight analysis in RM1-bearing mice. **(A)** Tumor entities from RM1-bearing mice at day 8 are shown for both control and Osthole-treated groups. **(B)** The subsequent growth curve documented the progressive increase in tumor volume over time (**P < 0.01). **(C)** A bar graph quantified the tumor weights, yielding a comparative assessment of the neoplastic burden in RM1 tumor-bearing mice (*P < 0.05).

**Figure 5:**
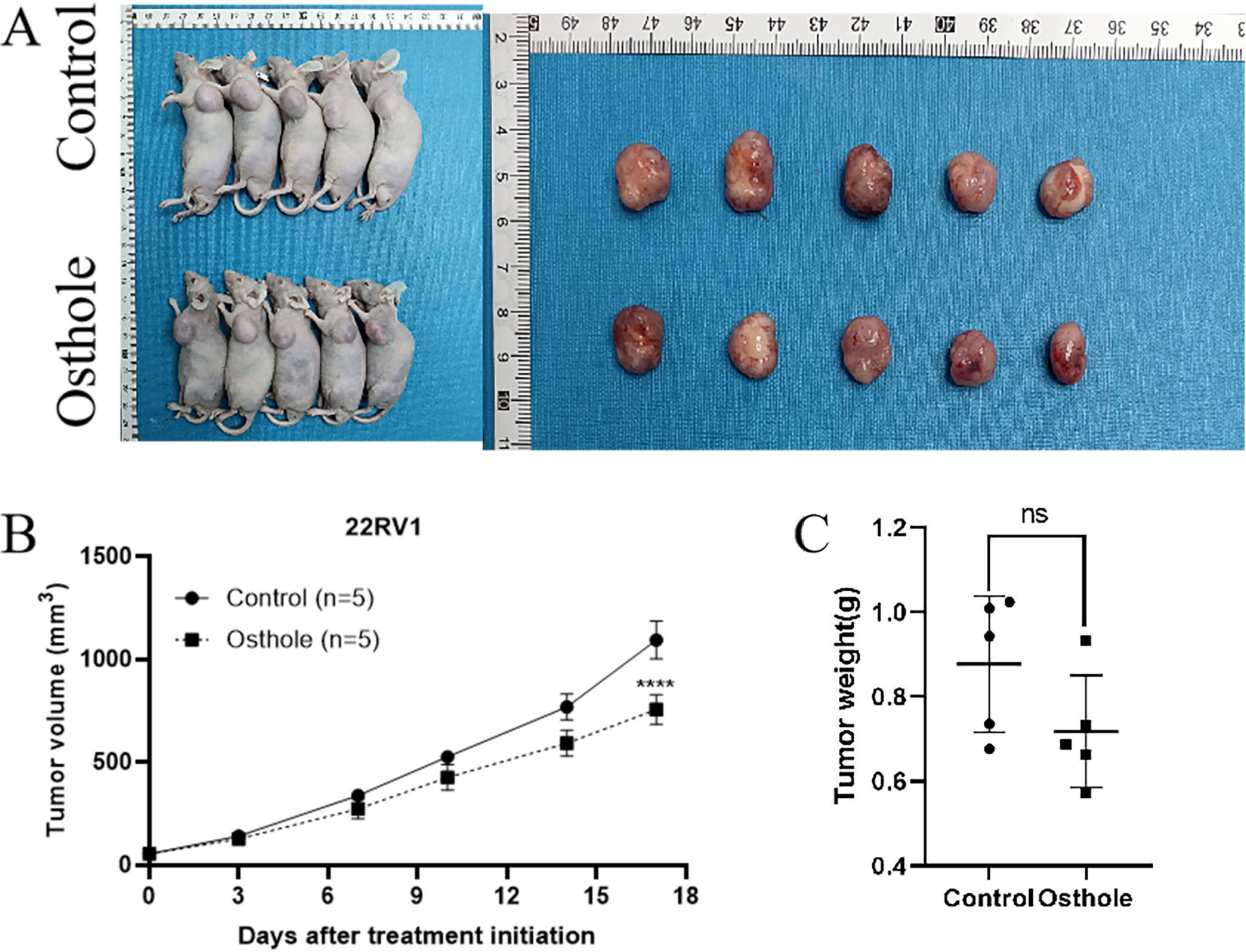
Tumor growth and weight analysis in 22RV1-bearing mice. **(A)** Tumor entities from 22RV1-bearing mice at day 17 are presented for both the control and Osthole-treated groups. **(B)** The growth curve on day 17 demonstrated a statistically significant increase in tumor volume over time in the control group (****P < 0.0001). **(C)** A bar graph quantified the tumor weights, providing a comparative assessment of the neoplastic burden in 22RV1 tumor-bearing mice.

For this study, we selected the human prostate cancer cell line 22RV1 and murine prostate cancer cell line RM1 to establish a CDX model for investigating prostate cancer progression. In view of the fact that DU145 and PC-3 are human-derived AR-negative prostate cancer cell lines, RM1 is a murine-derived AR-negative prostate cancer cell line, and 22RV1 is an AR-positive prostate cancer cell line, we selected one AR-positive cell line (22RV1) and one AR-negative cell line (RM1) for subsequent experiments. Additionally, the murine RM1 cell line was used to eliminate the potential confounding effects of spontaneous tumorigenesis in mice on the experimental outcomes. Osthole treatment significantly suppressed tumor growth in mice. Compared with the untreated control group, mice administered Osthole showed a markedly reduced rate of tumor volume expansion during the treatment period (*P* < 0.05)(**Figures 4A**, **5A**). Upon completion of the treatment regimen, the average tumor size in RM1 tumor-bearing mice treated with Osthole was significantly reduced compared to that in the control group (*P* < 0.01) (**Figures 4B, C**). Although a decreasing trend in tumor volume was observed in 22RV1 tumor-bearing mice subjected to Osthole treatment, the reduction in tumor weight was not statistically significant (**Figures 5B, C**). The lack of significance in 22RV1 xenograft tumor weight may reflect limitations of AR-positive cell models in immunodeficient mice.

### 3.5 Correlation between PRLR and prognosis in prostate cancer

**Figure 6:**
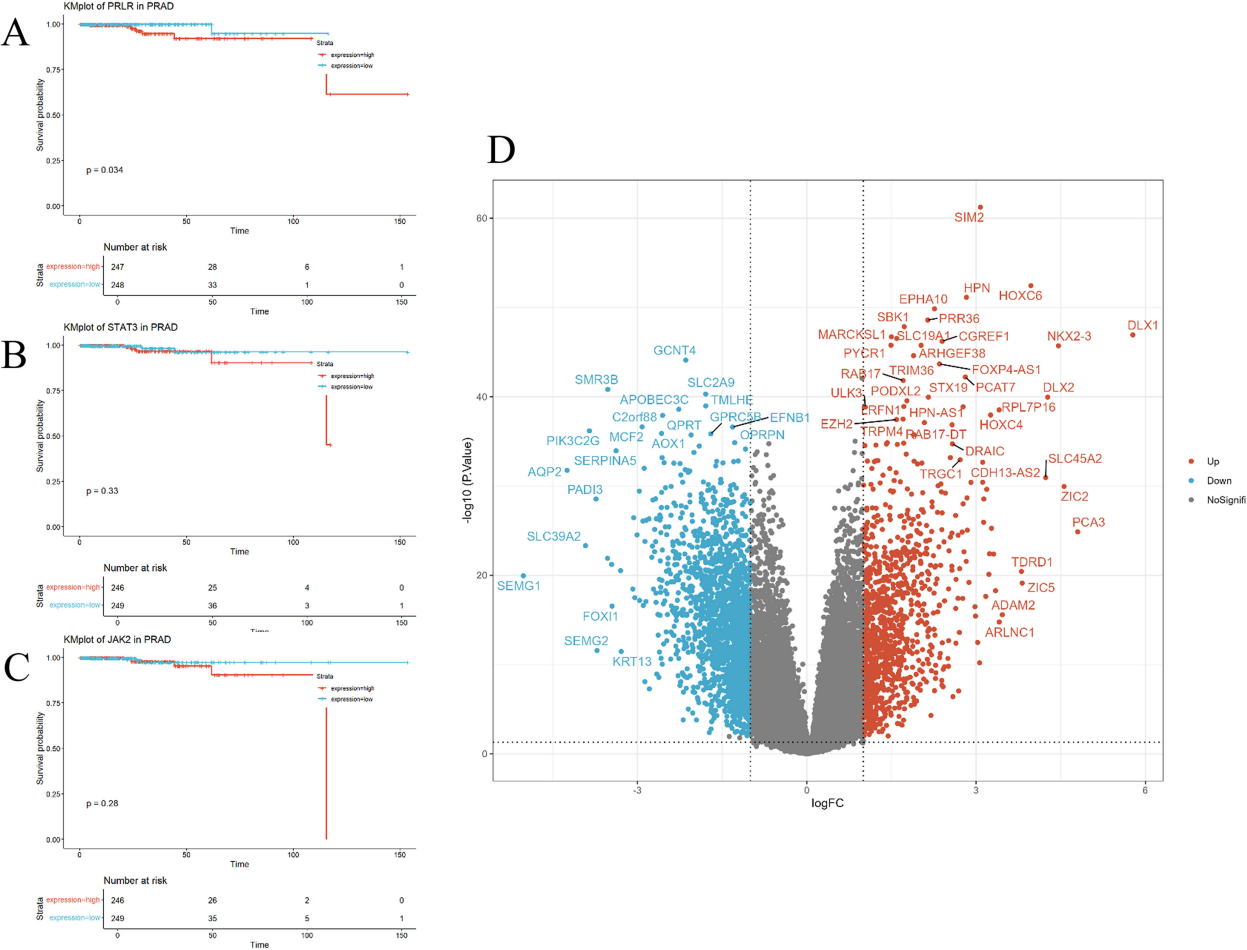
Correlation of PRLR, JAK2, and STAT3 genes with prognosis. **(A–C)** Kaplan-Meier survival curves for PRLR, JAK2, and STAT3 genes in prostate cancer patients. Survival curves for the high-expression group (red) and the low-expression group (blue) show distinct trends. High expression of the PRLR gene is significantly associated with worse survival outcomes (p = 0.034), whereas the expression levels of JAK2 and STAT3 genes are not significantly correlated with survival (p = 0.28 and p = 0.33, respectively). **(D)** Volcano plot illustrating the expression of differentially expressed genes in prostate cancer. The x-axis (logFC) denotes the log-fold change in gene expression, and the y-axis (-log10(P.Value)) denotes the negative log-transformed P-value. Red dots indicate the 1,087 significantly upregulated genes, blue dots indicate the 1,829 significantly downregulated genes, and gray dots represent the remaining genes that do not show significant changes. Out of a total of 32,151 genes analyzed, 30,235 genes are not significantly differentially expressed.

To investigate the correlation between PRLR and prostate cancer, we identified differentially expressed genes between prostate cancer patients and normal individuals from public databases. Bioinformatics analysis was then performed to assess the relationship between the expression levels of PRLR, JAK2, and STAT3 genes and the survival of prostate cancer patients. The results showed that high expression of the PRLR gene was significantly associated with poor survival outcomes, while JAK2 and STAT3 gene expression levels were not correlated with prognosis (**Figures 6A–C**). The volcano plot indicated significant upregulation of genes such as SIM2, HPN, and HOXC6 in prostate adenocarcinoma (PRAD), and significant downregulation of genes such as SLC39A2, SLC39A6, and SLC39A10 (**Figure 6D**).

**Figure 7:**
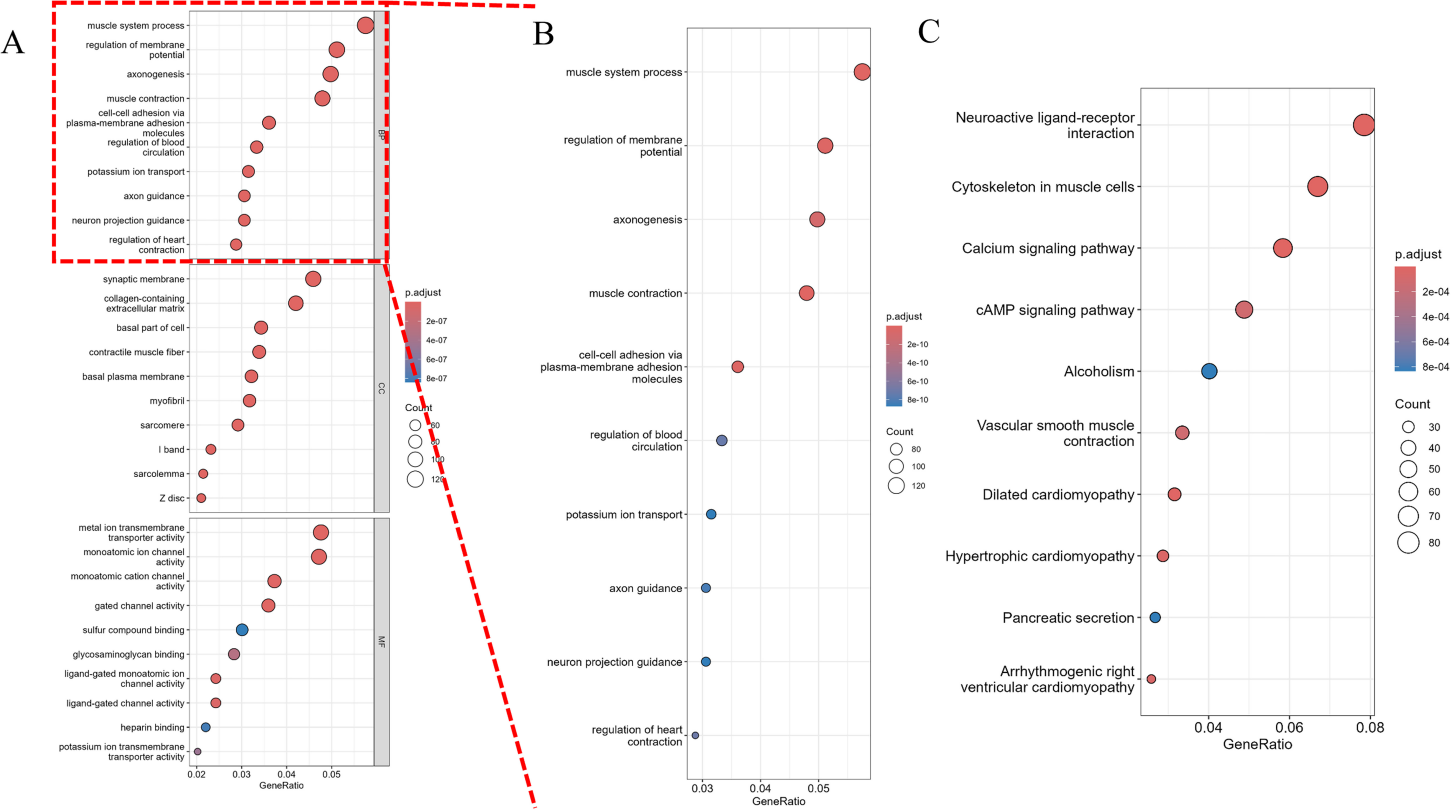
Pathway analysis of differentially expressed genes. **(A)** Gene Ontology (GO) analysis, including enrichment analysis of biological processes (BP), molecular functions (MF), and cellular components (CC). The x-axis represents the gene ratio (GeneRatio), and the y-axis lists the significantly enriched pathways. The size of the dots represents the gene count (Count), and the color intensity indicates the significance of the adjusted p-value (p.adjust), with darker colors representing smaller p.adjust values and higher significance. **(B)** An enlarged view of the biological processes (BP) section from the GO analysis, providing a detailed visualization of the enriched BP terms. The x-axis represents the gene ratio (GeneRatio), and the y-axis lists the significantly enriched BP pathways. The size of the dots represents the gene count (Count), and the color intensity indicates the significance of the adjusted p-value (p.adjust), with darker colors representing smaller p.adjust values and higher significance. **(C)** KEGG pathway analysis. The x-axis represents the gene ratio (GeneRatio), and the y-axis lists the significantly enriched pathways. The size of the dots represents the gene count (Count), and the color intensity indicates the significance of the adjusted p-value (p.adjust).

GO enrichment analysis (**Figure 7A**) revealed significant enrichment of biological processes such as “muscle system processes” and “regulation of membrane potential,” which are critical for tumor cell motility and ion channel-mediated signaling—processes frequently dysregulated in cancer cell invasion. These findings suggest that Osthole may disrupt prostate cancer cell migration by modulating genes involved in cytoskeletal dynamics and membrane potential homeostasis. An enlarged view of the biological processes section (**Figure 7B**) offered a detailed look at enriched BP terms like axon guidance. For KEGG pathway analysis, significant pathways including neuroactive ligand - receptor interaction, muscle cytoskeleton - related processes, and the calcium signaling pathway were detected (**Figure 7C**). These findings highlight the potential roles of the gene sets in biological functions and disease processes.

**Figure 8:**
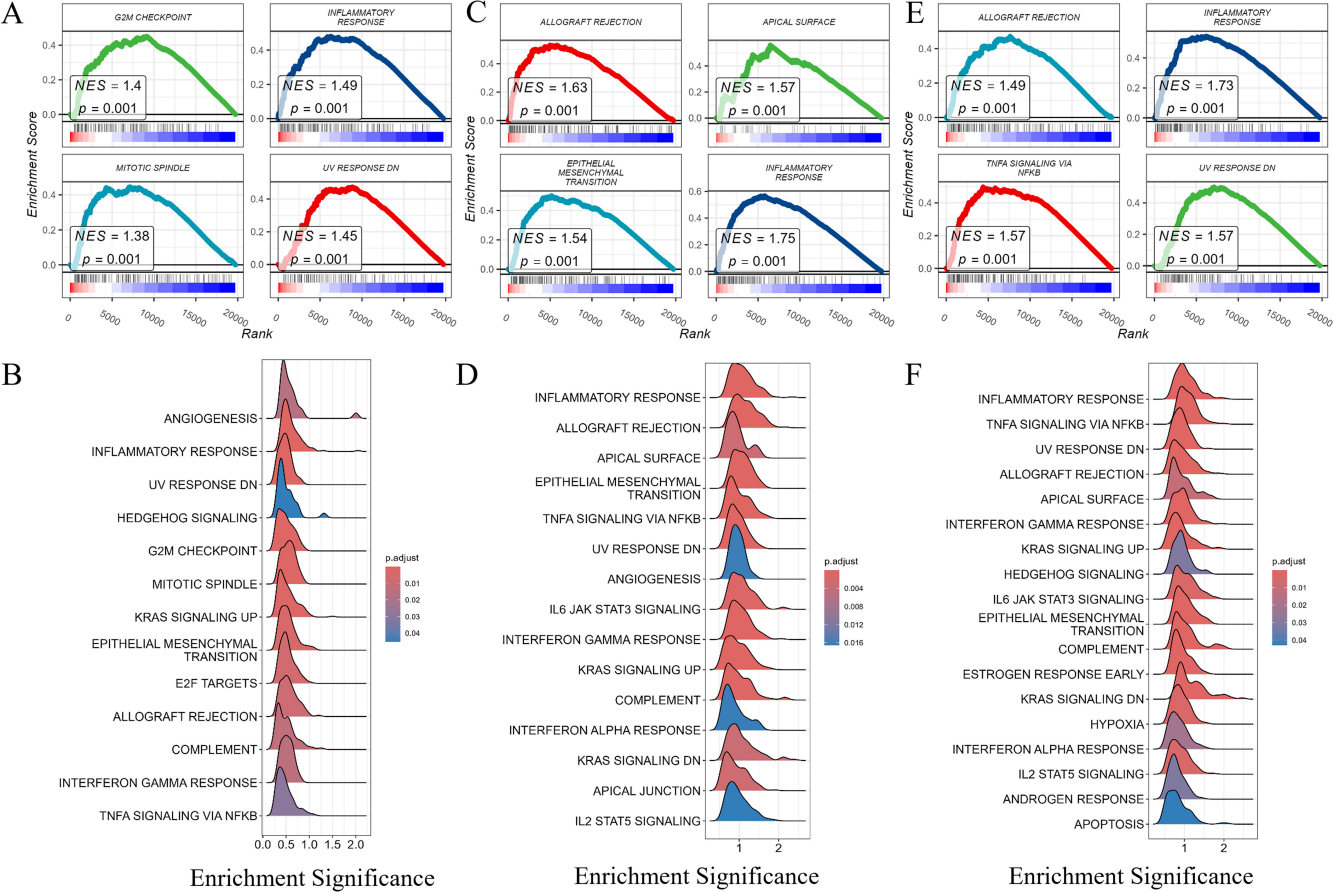
Gene set enrichment analysis (GSEA) of PRLR, STAT3, and JAK2. Gene set enrichment analysis (GSEA) of PRLR, STAT3, and JAK2. **(A, B)** GSEA of PRLR - associated pathways. Notably, the “G2M CHECKPOINT” and “INFLAMMATORY RESPONSE” pathways were significantly enriched, with normalized enrichment scores (NES) of 1.4 and 1.49, respectively. The size and color of the dots represent the gene ratio (GeneRatio) and the adjusted p - value (p.adjust), respectively. **(C, D)** GSEA of STAT3 - associated pathways. The “ALLOGRAFT REJECTION” and “EPITHELIAL-MESENCHYMAL TRANSITION” pathways were highly enriched, showing NES values of 1.63 and 1.54. Dot size and color correspond to GeneRatio and p.adjust, respectively. **(E, F)** GSEA of JAK2 - associated pathways. The “KRAS SIGNALING UP” and “IL6 JAK STAT3 SIGNALING” pathways were significantly enriched, with NES values of 1.49 and 1.57. The size and color of the dots reflect GeneRatio and p.adjust, respectively.

Finally, we conducted enrichment analysis of pathways associated with PRLR, JAK2, and STAT3 genes. The “G2M CHECKPOINT” (normalized enrichment score, NES = 1.4) and “INFLAMMATORY RESPONSE” (NES = 1.49) pathways were significantly enriched for PRLR-associated pathways (**Figures 8A, B**). The “ALLOGRAFT REJECTION” (NES = 1.63) and “EPITHELIAL - MESENCHYMAL TRANSITION” (NES = 1.54) pathways were highly enriched for STAT3 - associated pathways (**Figures 8C, D**). The “KRAS SIGNALING UP” (NES = 1.49) and “IL6 JAK STAT3 SIGNALING” (NES = 1.57) pathways were significantly enriched for JAK2 - associated pathways (**Figures 8E, F**). The results revealed significant enrichment of gene sets related to muscle system processes, regulation of membrane potential, and axonogenesis under disease conditions.

### 3.6 JAK2/STAT3 pathway regulation by osthole through PRLR interaction

**Figure 9:**
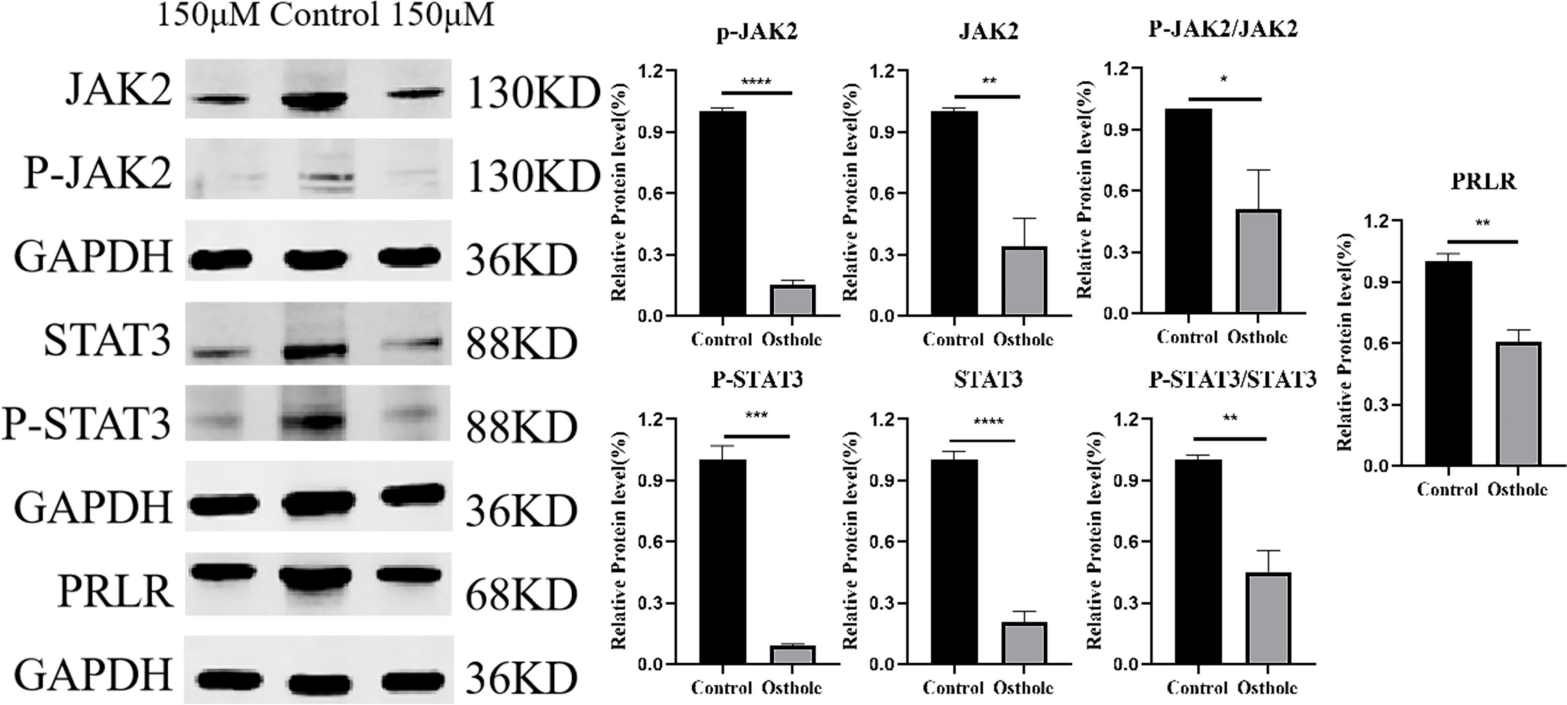
Western blot analysis. 150 μM Osthole significantly reduced the protein expression levels of PRLR, JAK2, and STAT3, as well as the phosphorylated forms of JAK2 and STAT3, and the ratio of phosphorylated to total JAK2 and STAT3 in 22RV1 cells. (*P<0.05, **P<0.01, ***P<0.001, ****P<0.0001).

To uncover the molecular underpinnings of the impact of Osthole on prostate cancer, this study utilized a western blot assay to methodically evaluate the expression levels of PRLR, as well as the expression and phosphorylation levels of JAK2 and STAT3 in the 22RV1 cell line. The selection of the 22RV1 cell line was driven by existing literature that reports the presence of PRLR, a factor deemed essential for delving into the molecular response to Osthole in prostate cancer (13). Western blot analysis revealed that, in contrast to the untreated control group, the protein expression level of PRLR in 22RV1 cells was downregulated after a 48-hour exposure to Osthole. Simultaneously, there was a notable reduction in the phosphorylation of JAK2 and STAT3 (**Figure 9**). These observations align with network pharmacology predictions and molecular docking results. By downregulating PRLR, Osthole disrupts the ligand-dependent activation of JAK2, leading to reduced STAT3 phosphorylation — a critical event in tumor cell proliferation and resistance to apoptosis.

## 4 Discussion

### 4.1 Clinical significance of osthole in prostate cancer therapy

Our findings indicate that Osthole markedly suppresses the proliferation and metastasis of prostate cancer cells, shows a tendency to induce apoptosis, and attenuates tumor growth in *in vivo* models. Prostate cancer is a global leading malignancy with rising incidence. Current therapies (e.g., surgery, chemotherapy) show limited efficacy in advanced stages and cause significant side effects (18). Therefore, the development of innovative pharmaceuticals is essential to bolster the therapeutic efficacy and elevate the living standards of affected individuals. Osthole, identified as a naturally occurring compound from a range of botanical sources, has recently demonstrated promising therapeutic potential in cancer research. It has been shown to significantly inhibit the growth of multiple cancer cell types, notably those associated with gastric cancer (6). Owing to its diverse biological functions, including anti-inflammatory, antioxidant, and anti-neoplastic capabilities, Osthole is a promising candidate for the development of new prostate cancer treatments. Compared to existing therapies, Osthole offers distinct advantages. Current prostate cancer treatments include surgery, radiotherapy, chemotherapy, and endocrine therapy. However, these are often associated with significant side effects. For instance, surgery can lead to urinary incontinence and erectile dysfunction, while chemotherapy may cause nausea, vomiting, hair loss, and bone marrow suppression (19). Moreover, the efficacy of these treatments for advanced or metastatic prostate cancer is limited. As a natural compound, Osthole has multi - target and multi - pathway effects, along with good tolerability and low toxicity (15). Our *in vitro* and *in vivo* experiments showed significant anti - prostate cancer activity with no obvious toxic reactions, suggesting Osthole may be a safer and more effective treatment option.

### 4.2 Network pharmacology reveals PRLR/JAK2/STAT3 as a novel target

This article initially explores the potential of Osthole to inhibit prostate cancer through network pharmacology analysis, utilizing multiple public databases to investigate possible targets and signaling pathways. By mapping the interactive network of the compound with biological entities, network pharmacology offers an encompassing view of Osthole’s molecular targets, its associated signaling cascades, and their intricate relationships within the biological context. This comprehensive method of research not only improves the accuracy and efficiency of drug development but also provides scientific evidence for the clinical application of the drug. We have identified targets such as AKT1, TNF, IL6, STAT3, and CTNNB1 in Osthole’s inhibition of prostate cancer, along with pathways including the TNF signaling pathway, the Interleukin-6-17 (IL-6-17) signaling pathway, and the prolactin signaling pathway. Studies have indicated that Osthole can inhibit prostate cancer by suppressing the TGF-β/Akt/MAPK pathway (9), which is consistent with our network pharmacology findings. However, our research also revealed that Osthole can exert anti-prostate cancer effects by binding to PRLR, thereby inhibiting the JAK2/STAT3 pathway. Prolactin (PRL), a polypeptide hormone secreted by the anterior pituitary gland in mammals, is a member of the growth hormone family. Its primary physiological role is to stimulate the growth and development of mammary glands and regulate milk production during late pregnancy and lactation (20). In addition, PRL is involved in the regulation of the reproductive system, immune system, and various other physiological processes in the body (21). Studies have demonstrated that PRLR promotes the growth of prostate cancer cells by regulating multiple signaling pathways. Specifically, upon binding of PRL to PRLR, downstream signaling pathways such as JAK-STAT, AKT, and MAPK are activated; this activation leads to increased cell proliferation, survival, and migration (22). Additionally, the interaction between PRLR and the androgen receptor plays a critical role in the progression of prostate cancer. This crosstalk may enhance the sensitivity of tumor cells to androgens, thereby facilitating tumor growth and metastasis (19). However, to date, no studies have reported that Osthole can inhibit prostate cancer growth via PRLR. Thus, our study holds significant relevance.

### 4.3 Experimental validation of osthole’s anti-cancer effects

Based on the network pharmacology forecast, we verified the effects of Osthole on prostate cancer cell proliferation through cellular experimentation. By leveraging CCK8 and Transwell invasion assays for cell viability assessment, we established the growth-inhibiting role of Osthole in bladder cancer cells. While earlier research employed DU145 and PC3 human bladder cancer cell lines as models to study the suppressive effects of Osthole on prostate cancer (9), our study examined a more extensive range of prostate cancer cell lines. The experimental outcomes with the DU145 and PC3 cell lines were consistent with the findings reported in the aforementioned literature. However, the effects of Osthole on the 22RV1 cell line have not been documented in the existing literature to date. Therefore, our experiments have broadened the horizons of research in this area, and we observed that cells treated with Osthole exhibited nuclei that were smaller in size, deeply stained, and disorganized in arrangement, suggesting their potential pro-apoptotic capabilities.

### 4.4 Molecular simulations confirm high-affinity PRLR binding

Molecular docking studies were conducted to elucidate the molecular mechanisms underlying the action of Osthole. The results demonstrated robust binding interactions between Osthole and both the PRLR and STAT3 proteins, suggesting a theoretical framework whereby Osthole could potentially suppress the JAK2/STAT3 signaling pathway through its interaction with PRLR. Molecular dynamics simulation results revealed that the PRLR-Osthole complex reached a relatively stable state after 20 ns, with structural parameters such as RMSD, Rg, and SASA remaining within stable ranges. The protein backbone structure was compact with low fluctuation, and the small molecule Osthole was stably positioned in the binding pocket without detachment. Hydrogen bonds and hydrophobic interactions collectively maintained binding stability, and binding free energy analysis also demonstrated favorable affinity. Osthole’s high-affinity binding to PRLR (ΔG = −19.14 kcal/mol) may induce conformational changes that promote receptor degradation, leading to reduced PRLR protein levels. In summary, the complex system exhibited good thermodynamic stability and binding properties during the simulation, holding potential for further development as a potential inhibitor. This suggests that Osthole possesses the ability to inhibit both the proliferation and metastatic capabilities of breast cancer cells, primarily through the suppression of the JAK2/STAT3 signaling cascade (17). However, the analogous potential of Osthole to exert inhibitory effects in prostate cancer, along with the intricate mechanisms driving this process, remains to be more rigorously explored and empirically confirmed. The choice of PRLR as the primary target is based on several considerations. Network pharmacology predicts an Osthole - PRLR interaction, confirmed by molecular docking. Evidence also highlights PRLR’s role in various cancers, acting through pathways like JAK2/STAT3 to regulate tumor cell proliferation, survival, invasion, and immune evasion (8). While Osthole might influence other pathways, such as PI3K/AKT (15), our analysis and experiments emphasize the PRLR - JAK2/STAT3 pathway’s key role in Osthole’s anti - prostate cancer effects. Further research is needed to explore if Osthole affects other pathways.

### 4.5 Therapeutic advantages over existing pathway inhibitors

In prostate cancer treatment, the PRLR and JAK2/STAT3 signaling pathways are crucial targets. Current therapies include specific inhibitors to block these pathways. For example, a study reported an immunotoxin targeting PRLR to enhance tamoxifen sensitivity and chemotherapy efficacy in breast cancer (10). This research showed that tamoxifen upregulates PRLR in breast cancer cells, leading to resistance. An immunotoxin designed against PRLR restored tamoxifen sensitivity and improved chemotherapy outcomes *in vitro* and *in vivo*. However, clinical application of PRLR and JAK2/STAT3-targeted therapies for prostate cancer is still limited, partly due to severe side effects like immunosuppression and cytotoxicity (19). Osthole has shown significant inhibition of the PRLR and JAK2/STAT3 pathways. Unlike traditional single-target inhibitors, Osthole has multi-target properties, which may give it an edge in treating complex diseases like cancer. For instance, Osthole not only suppresses the PRLR and JAK2/STAT3 pathways but may also produce synergistic effects by modulating other pathways like PI3K/AKT (15), potentially reducing drug resistance.

### 4.6 PRLR’s prognostic value and inflammation link via TCGA analysis

Although Osthole shows significant anti-prostate cancer activity *in vitro* and *in vivo* with no obvious toxicity, its potential off-target effects need further study. With its multi-target nature, Osthole may interact with various proteins, causing unexpected biological effects. While this multi-target action enhances therapeutic effects, it may also lead to off-target effects. Preclinical studies should assess these effects in different cells and animal models to ensure Osthole’s safety and efficacy. To enhance Osthole’s therapeutic effects and reduce off-target effects, advanced drug delivery systems like nano-encapsulation or liposomal delivery could be used to improve its tumor targeting and stability (4). Future research should also explore Osthole’s molecular mechanisms, particularly in inflammatory responses, to better understand its therapeutic potential.

In this study, we analyzed RNA sequencing data of prostate cancer patients from the TCGA database and identified that high PRLR expression was significantly associated with poor overall survival, suggesting its potential as a prognostic biomarker for prostate cancer. Previous studies have indicated that the association between PRLR and inflammatory response may be a key mechanism through which PRLR contributes to tumor progression. Chronic inflammation, as a critical component of the tumor microenvironment, can promote tumor progression by enhancing angiogenesis, immune evasion, and cell proliferation (23). Therefore, PRLR may influence patient prognosis by activating inflammation-related pathways that reshape the tumor microenvironment.

Although JAK2 and STAT3 expression levels were not significantly correlated with prognosis in this study, it is noteworthy that these two genes have been implicated in tumor microenvironment regulation in other cancer types. For instance, JAK2 has been associated with tumor mutational burden in prostate cancer (24), and STAT3 is recognized as a pivotal regulator in inflammatory and tumor signaling networks (25). Thus, JAK2 and STAT3 may not directly contribute to prostate cancer progression but could modulate the immune tumor microenvironment, thereby promoting disease development. Further analysis revealed significant enrichment of PRLR, JAK2, and STAT3 in the inflammatory response pathway, indicating that inflammation may serve as a central pathway mediating the combined effects of these genes. The inflammatory response pathway not only directly impacts cancer cell proliferation and survival but also promotes epithelial-mesenchymal transition (EMT) and immune suppression, driving tumor progression (26). From a clinical perspective, the prognostic potential of PRLR provides a promising tool for risk stratification and personalized treatment of prostate cancer patients. Furthermore, the shared enrichment of PRLR, JAK2, and STAT3 in the inflammatory response pathway suggests that targeting this pathway could be a viable therapeutic strategy to inhibit their combined effects. For example, anti-inflammatory therapies could simultaneously mitigate PRLR’s role in inflammatory responses and suppress the deterioration of the tumor microenvironment.

### 4.7 Mechanistic confirmation: osthole suppresses PRLR/JAK2/STAT3

To substantiate our hypothesis empirically, we performed western blot analysis, which demonstrated a marked reduction in the expression levels of PRLR in the 22RV1 prostate cancer cell line following treatment with Osthole. This significant downregulation of the PRLR protein indicates that Osthole may modulate the expression of key receptors involved in prostate cancer progression. Subsequent treatment with Osthole resulted in a notable reduction in the phosphorylation status of both JAK2 and STAT3 proteins, implying an inhibitory effect on the JAK2/STAT3 signaling pathway. Studies have shown that the expression of PRLR is not ubiquitous among prostate cancer cell lines (19), implying that the anticancer action of Osthole could be mediated through alternative pathways in addition to PRLR. The JAK2/STAT3 pathway is a prevalent signaling cascade that facilitates the transduction of signals from the extracellular milieu to the nucleus upon the binding of cytokines and growth factors to specific cell surface receptors (13). Under normal conditions, the pathway is activated when prolactin, growth factors such as EGF, and cytokines, predominantly of the IL-6 family, bind to the extracellular domains of their respective receptor tyrosine kinases (RTKs) (14). This signaling pathway is tightly regulated and serves a multitude of biological functions, including cell proliferation, differentiation, and apoptosis (27). However, aberrant activation of the JAK2/STAT3 pathway is frequently detected in various tumors and has been implicated in the genesis, angiogenesis, and metastasis of many cancers (28). Additionally, the JAK2/STAT3 pathway can interact with other pathways, such as the NF-κB and FOXO pathways, which are closely associated with cancer development (29, 30).

### 4.8 Oncogenic role of JAK2/STAT3 in prostate cancer

In our detailed study of the effects of Osthole on prostate cancer, we found that it could suppress tumor growth and metastasis by targeting the JAK2/STAT3 pathway. This supports the accuracy of network pharmacology predictions and suggests the potential of Osthole as a treatment for prostate cancer. Further research could lead to more effective and safer therapies for such patients. Future research should further investigate the molecular mechanisms of PRLR within the inflammatory response pathway, with a particular focus on its potential synergistic interactions with JAK2 and STAT3. Although JAK2 and STAT3 did not show significant prognostic associations in the current analysis, functional experiments are warranted to validate their roles in inflammation-driven tumor progression. These findings could provide valuable insights into the development of anti-inflammatory strategies and targeted therapies, offering new directions for therapeutic intervention in prostate cancer.

### 4.9 Translational implications and future directions

Studies have shown that osthole has no significant toxicity to normal skin fibroblasts, hepatic stellate cells and normal neural stem cells, but no research has yet proved that it has no significant toxicity to normal prostate cancer cells (31–33), further validate the *in vitro* anti-cancer potential of osthole. To translate these preclinical findings into clinical benefits, several steps are essential. First, the bioavailability and pharmacokinetics of Osthole need to be optimized through advanced drug formulation strategies, such as nano - encapsulation or liposomal delivery, to enhance its stability and tumor - targeting efficiency. Second, given the promising results in prostate cancer models, well - designed phase I clinical trials should be initiated to assess the safety and tolerability of Osthole in prostate cancer patients. Subsequent phase II and III trials can then evaluate its efficacy in combination with or compared to standard therapies. Additionally, biomarker - driven patient selection, such as PRLR expression levels, may further enhance the therapeutic response and personalized treatment outcomes. The multi - target nature of Osthole also presents an opportunity to develop it as part of a combination therapy regimen, potentially reducing drug resistance and improving patient survival. Overall, our study provides a solid foundation for the further clinical development of Osthole as a novel therapeutic agent for prostate cancer.

## 5 Conclusion

In conclusion, this study validated the pharmacological mechanism of Osthole in prostate cancer using network pharmacology predictions and *in vivo* and *in vitro* studies. This suggests that Osthole inhibits the progression of prostate cancer by targeting the JAK2/STAT3 pathway. Therefore, Osthole is a potential drug for the treatment of prostate cancer.

## Data Availability

The original data analyzed in this study are publicly available from the Genomic Data Commons (GDC) portal (link: https://portal.gdc.cancer.gov/) under the TCGA-PRAD project.
